# Conjugation of Soybean Proteins 7S/11S Isolate with Glucose/Fructose in Gels through Wet-Heating Maillard Reaction

**DOI:** 10.3390/gels10040237

**Published:** 2024-03-29

**Authors:** Jalal Ud Din, He Li, You Li, Xinqi Liu, Sam Al-Dalali

**Affiliations:** 1 Key Laboratory of Geriatric Nutrition and Health, Beijing Technology and Business University, Ministry of Education, Beijing 100048, China; 2 National Soybean Processing Industry Technology Innovation Center, Beijing 100048, China; 3 Department of Food Science and Technology, Faculty of Agriculture and Food Science, Ibb University, Ibb 70270, Yemen; 4 School of Food and Health, Guilin Tourism University, Guilin 541006, China

**Keywords:** Maillard reaction, 7S protein, 11S protein, glucose, fructose, soybean

## Abstract

Conjugation with glucose (G) and fructose (F) via the Maillard reaction under the wet-heating condition is a natural and non-toxic method of improving the technological functions of 7S/11S proteins in different kinds of gels. It may be used as an affordable supply of emulsifiers and an excellent encapsulating matrix for gels. This study aimed to create a glucose/fructose-conjugated 7S/11S soy protein via the Maillard reaction. The conjugation was confirmed by determining the SDS-PAGE profile and circular dichroism spectra. In addition, these conjugates were comprehensively characterized in terms of grafting degree, browning degree, sulfhydryl content, surface hydrophobicity (H_0_), and differential scanning calorimetry (DSC) through various reaction times (0, 24, 48, and 72 h) to evaluate their ability to be used in food gels. The functional characteristics of the 7S/11S isolate–G/F conjugate formed at 70 °C, with a high degree of glycosylation and browning, were superior to those obtained at other reaction times. The SDS-PAGE profile indicated that the conjugation between the 7S and 11S proteins and carbohydrate sources of G and F through the Maillard reaction occurred. Secondary structural results revealed that covalent interactions with G and F affected the secondary structural components of 7S/11S proteins, leading to increased random coils. When exposed to moist heating conditions, G and F have significant potential for protein alteration through the Maillard reaction. The results of this study may provide new insights into protein modification and establish the theoretical basis for the therapeutic application of both G and F conjugation with soy proteins in different food matrixes and gels.

## 1. Introduction

In various parts of China, soybeans (*Glycine max.* L.) have been cultivated and incorporated into local cuisine. It is commonly used to produce dishes such as tempeh, natto, fermented dark soybeans, fermented soybean pastes, soy sauce, and fermented soybean curd. Soybeans are a balanced source of essential nutrients, containing protein, dietary fiber, and several bioactive phytochemicals. This versatile food is highly valued in China for its various health benefits, such as detoxification, anti-inflammatory and antitumor effects, cholesterol-lowering, and diuretic properties [1].

Soy protein is widely used in many food products and gels as a major functional food ingredient; it is a valuable source of protein that is of excellent quality and contains all the necessary amino acids [2]. Soy products contain advantageous constituents, such as soy isoflavones, which have demonstrated the ability to protect against cardiovascular disease [3]. Soy proteins are also the focus of much research due to their positive functional properties [4]. To improve the nutritional value and biological functions of soy protein and broaden its potential applications, one possible approach is to modify its natural solid structure and make it more flexible to be used in gel matrixes [5].

Soy protein isolates (SPIs) are high-quality plant proteins obtained from soybean meal that have been defatted using mild temperatures [6]. The protein constituents of SPI can be classified into 2S, 7S, 11S, and 15S groups, based on their sedimentation coefficients at pH 7.6 and ionic strength of 0.5 mol/L. Out of these components, 7S (β-conglycinin) and 11S (glycinin) constitute 80% of the overall content [7,8].

Food’s structure is largely composed of proteins. Meat and dairy products are dietary sources of protein and nutrients (minerals, bioavailable heme iron, zinc, vitamin B, etc.). Although dairy proteins, like casein and whey protein, are frequently used to structure food, there has been a rush to develop plant proteins as a substitute for animal protein due to the growing need for diets that are more environmentally friendly and sustainable [9].

By 2100, it is projected that the global population will reach 11.2 billion, which would exert significant pressure on food resources around the globe. Furthermore, this will give rise to issues concerning sustainable development, public health, the environment, and animal welfare. The popularity of plant-based meat replacements is growing due to its promotion of improved health and environmental conservation [10].

Maillard reactions occur in food products when exposed to high temperatures during processing (e.g., roasting, cooking, extruding, etc.) or when stored for long periods at room temperature. These reactions can also occur during external transport when the temperature inside shipping containers can rise to 70 °C [11,12]. The conjugation of reducing sugars with ε-amino groups of amino acids or proteins via Maillard reactions has received considerable interest, particularly because of its ability to improve emulsifying properties in gels and thermal stability by creating covalently coupled products [13]. This reaction is usually performed without the use of additional chemicals. To study protein glycosylation, researchers often use monosaccharides such as glucose and fructose in their studies [14,15].

Glycation reactions occur when the reducing end of sugars binds to the terminal proteins and side groups of proteins. The process of attaching carbohydrates to proteins enhances their capacity to dissolve in solutions with adverse conditions such as a low pH and high ionic strength. This, in turn, improves the functionality of proteins and resolves the denaturation issues that occur in low-pH or high-ionic-strength environments. The investigation of the impact of glucose on the secondary and tertiary/quaternary structures of proteins has gained significant attention in current research, particularly in relation to the sugar content in dietary proteins [16].

The process of attaching polysaccharides to proteins, known as grafting, can enhance the structural stability of proteins by augmenting their resistance to spatial forces and electrostatic repulsion [17]. Furthermore, glycoside enhances the quantity of hydroxyl groups in proteins, resulting in heightened solubility and surface hydrophilicity, as well as enhanced emulsifying characteristics [18].

Maillard reaction gels are formed through the chemical bonding of proteins and monosaccharides. The gels are composed of distinct combinations of monosaccharide proteins [19]. The protein–sugar interactions encompass hydrophobic and electrostatic interactions, as well as covalent and hydrogen bonds. Protein–sugar conjugation primarily takes place via covalent linkages [20]. During the Maillard reaction, alterations in the protein structure might result in the exposure of intramolecular sulfhydryl groups on the surface. Heating can disrupt disulfide bonds, hence promoting the formation of sulfhydryl groups. Furthermore, the process of thermal denaturation increases the effectiveness of the sulfhydryl group on the surface of the molecule, enabling it to engage in the reaction of exchanging sulfhydryl/disulfide bonds [21].

When the temperature exceeds 65 °C, the hydrophobic and sulfhydryl residues become exposed due to the unfolding of the globular protein. This exposure promotes protein aggregation by facilitating hydrophobic cross-linking and disulfide bond formation. The production of a gel with suitable protein concentrations is achieved through the creation of a three-dimensional network of protein particles that fills the full volume of the system [22]. Furthermore, the 7S/11S protein offers a range of physical, chemical, and structural characteristics, including gelling, emulsifying, foaming, and flavor-binding, which enhance the sensory qualities, texture, and rheological aspects of food items. Nevertheless, the application of 7S/11S and G/F conjugates in the industrial sector is restricted due to their inadequate solubility, poor emulsification stability, and propensity to coagulate under specific processing conditions, such as an elevated ionic strength, temperature, or alkaline pH, have been identified [23].

The purpose of this investigation was to evaluate the potential of glucose/fructose-conjugated soy protein 7S/11S through Maillard reaction for use in gels and food industry products. These data will serve as a basis for determining the potential applications of soy protein in food products. This study will provide insight into the use of the Maillard reaction with glucose/fructose conjugation with 7S/11S soy protein, which can significantly improve its functional properties, making it suitable for various food applications [24,25,26,27,28,29,30].

## 2. Results and Discussion

### 2.1. Determination of the Grafting Degree and Browning Degree

The Maillard reaction is a complex process with several intermediates and sophisticated end products that was influenced by a variety of circumstances [31]. The extent of the Maillard reaction can be assessed by measuring the active amino content, which is quantified as the grafting degree (GD) [32]. The amino group of the protein’s amino acid side chain and the carbonyl group at the end of monosaccharide molecules were the main participants in the 7S/11S glycosylation process. The degree of glycosylation grafting reaction can be assessed using the GD. Therefore, alterations in the free amino group on the 7S/11S with G/F molecular chain may have a similar effect.

**Figure 1:**
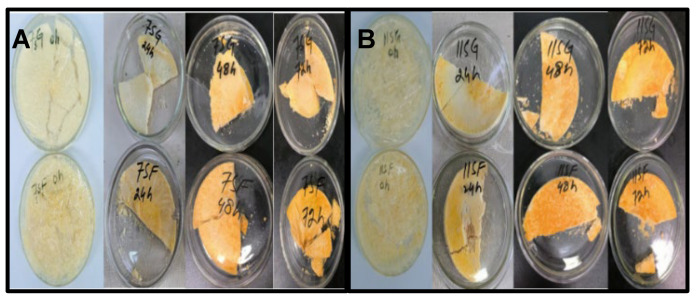
Effects of reaction time on GD and browning degree of 7S/11S with G/F conjugates: (**A**) conjugation of 7SG and 7SF; (**B**) conjugation of 11SG and 11SF over 0, 24, 48, and 72 h.

**Figure 2:**
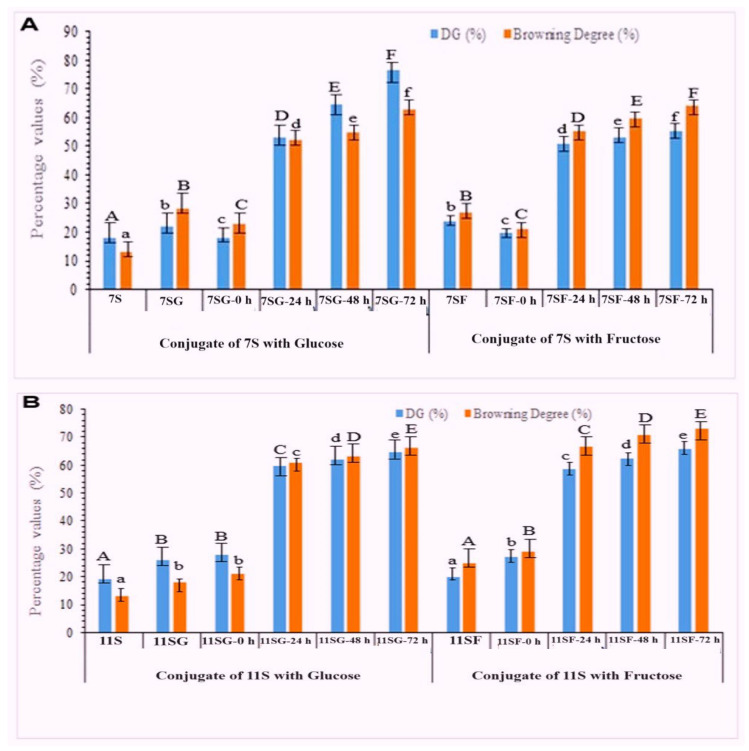
Development of browning of the 7S/11S with glucose/fructose conjugates as measured with absorbance at 420 nm as a function of reaction time: (**A**) conjugation of 7SG and 7SF; (**B**) conjugation of 11SG and 11SF over different reaction times. Tukey’s pairwise comparison revealed that values that do not share the same letter (superscript) are significantly different.

Figure 1 and Figure 2 display the browning activity and GD of 7S/11S with G/F conjugates produced under various treatment settings. Over time, the browning activity of 7S/11S with G/F and the GD values of the 7S/11S with G/F conjugates were both greater. The aldehyde group undergoing reduction must come into contact with the amino group present on the protein chain to start the grafting process between 7S/11S with G/F. Although not all the amino groups on 7S/11S with G/F chains are exposed at the start of the reaction, incubation can help increase this exposure [33]. The conjugation of 7S/11S with G/F content increased rapidly from time zero to 22 h and then became nearly constant at 65% and 70%. However, the Maillard reaction for too long a time might cause the polymerization of protein and hide some of the reactive groups, which resulted in a decrease in the grafting degree. The initial Maillard condensation of aldehyde with an amine is the first step in a lengthy series of reactions that eventually produce crosslinked proteins and brown coloration via rearrangements and fragmentations. Some studies showed that the loss of active amino group content during the Maillard reaction might be due to the generation of a Schiff base [34]. Moreover, 7S/11S with G/F often browned more quickly than the original 7S and 11S shown in Figure 1A,B, which might be due to reducing the end carbonyl group of the sugar, and the free amino groups of protein may react to intensify the red color. The increased absorbance value that comes with browning can be utilized as a measurement for the Maillard reaction’s more advanced phases of browning. [35]. The increased degree of browning of 7S/11S with G/F supported the existence of more advanced glycosylation end products and maybe even melanoidins.

### 2.2. Sulfhydryl Content of the Protein Surface

Important chemical connections known as sulfhydryl and disulfide bonds stabilized the shape of the molecules of protein and were crucial for the functional characteristics of proteins, including their ability to foam and emulsify in gels [36]. Research revealed that two factors contributed to the variations in the protein molecule’s free sulfhydryl group concentration. One factor was that an outside force caused the protein’s structure to unfold, exposing the original sulfhydryl inside the molecule. Another scenario could indicate that the disulfide link broke as a result of the protein subunits dissociating, releasing a fresh free sulfhydryl group [34]. The denatured state of proteins was reflected in the variations in the free sulfhydryl concentration.

**Figure 3:**
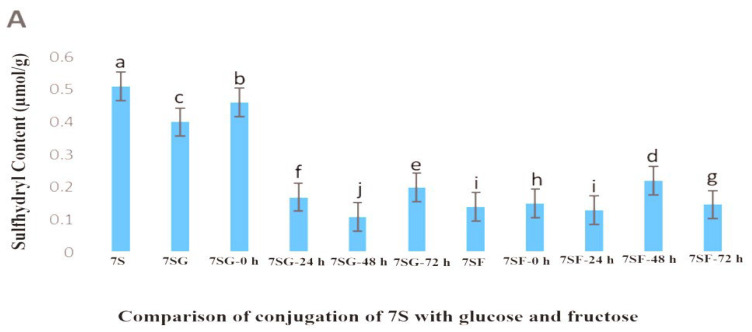

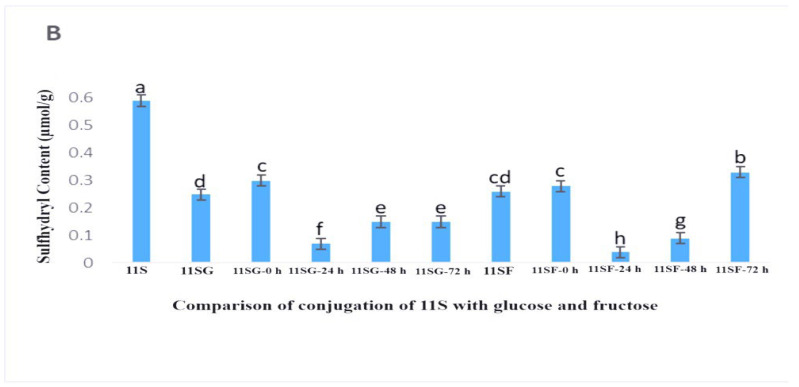
Reduction of the sulfhydryl content of the 7S/11S with glucose/fructose conjugates at different reaction times: (**A**) conjugation of 7SG and 7SF; (**B**) conjugation of 11SG and 11SF over different reaction times. Tukey’s pairwise comparison revealed that values that do not share the same letter (superscript) are significantly different.

Figure 3 illustrates the fact that the primary reasons for the lower sulfhydryl content within the 7S/11S protein, observed at 48 and 72 h following heating in comparison to the native 7S/11S, comprised protein denaturation and aggregation. The soy protein isolates exhibited a reduced sulfhydryl concentration because free-SH was partially entrenched in the protein’s hexamer (7S/11S) configuration and was unable to be fully exposed in its natural condition [37]. The percentage of denatured samples elevated considerably (*p* < 0.05) with longer reaction times, reaching a maximum after 48 h. This could be attributed to protein structural changes that reached the 7SG’s spatial arrangement, as well as exposing the sulfhydryl group, which was initially rooted in the molecule’s interior, increasing its flexibility. The creation of disulfide bonds within or between polypeptides may have contributed to the content decrease whenever the period of reaction was longer than 48 h. Therefore, even though the result was lower than that of the native 7S/11S, it was not surprising because it showed a rising pattern in the amount of sulfhydryl concentration on the outer surface of the protein. Other research [38] determined the sulfhydryl content of soy protein isolates–glucose conjugates and analyzed the fact that free-SH groups were maximum at 5 h and then decreased, and it explained the same reason, which was consistent with our results in this study.

### 2.3. SDS-PAGE Analysis

**Figure 4:**
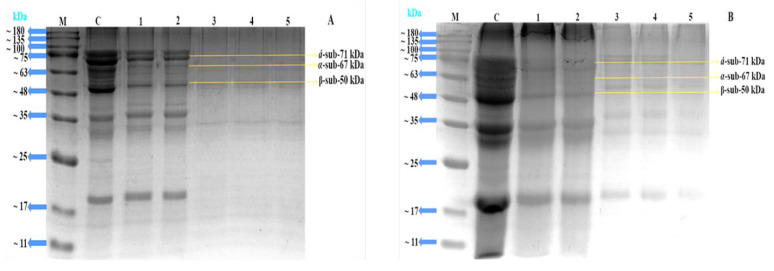
SDS-PAGE profile of 7S with glucose and fructose. Lane M: standard molecular weight marker; C: 7S protein; 1: 7SG; 2: 0 h; 3: 24 h; 4: 48 h; 5: 72 h; (**A**) the conjugation of 7S with glucose; and (**B**) the conjugation of 7S with fructose.

Protein subunits’ molecular weight diversity is often examined using SDS-PAGE. In this work, the conjugates’ glucose was dyed using the Schiff reagent along with protein staining. SDS-PAGE was used to confirm the covalent linking among 7S/11S with glucose and fructose because of the generation of high-molecular-weight mixtures [39]. It is shown in Figure 4A,B that certain wider and darker stripes had emerged in lanes 1–3. The color of the strip became shallower, while the response time rose, signifying the creation of aggregates via heating. These phenomena might be explained by the SPI subunits reacting with glucose and fructose to create a macromolecular polymer. Subsequently, a protein’s increased molecular weight was believed to serve as a significant indicator of the reaction’s conjugate production [40]. Simultaneously, Figure 4 displays the unprocessed SPI subunit bands, whereas each of the three 7S subunits is represented with α′, α, and β. The glycoprotein’s α′ and α subunit bands progressively grew light in color as the reaction time increased, suggesting that the protein’s structure was altered throughout the Maillard reaction. These phenomena aligned with the earlier findings of [41].

**Figure 5:**
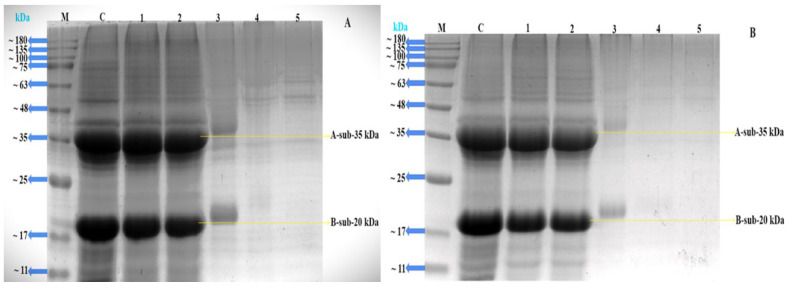
SDS-PAGE profile of 11S with glucose and fructose. Lane M: standard molecular weight marker; C: 11S protein; 1: 11SG; 2: 0 h; 3: 24 h; 4: 48 h; and 5: 72 h; (**A**) the conjugation of 11S with glucose; and (**B**) the conjugation of 11S with fructose.

It is shown in Figure 5A,B that certain wider and darker stripes had developed in lanes 1 and 2. The color of the strip became shallower as the impact of time rose, signifying the creation of aggregates via heating. This phenomenon might be explained by the SPI subunits reacting with glucose and fructose to create a macromolecular polymer. The subunit patterns of the untreated 11S are shown as well in Figure 5, where A and B represent the two distinct subunits of the 11S with sizes of 35 kDa and 20 kDa, respectively. The A and B distinct subunit bands of the 11S protein progressively became shallower as the reaction’s duration time increased, showing that the protein underwent structural changes during the Maillard reaction. These incidents were more noticeable regardless of the incubation time. Based on other studies [42,43], we may thus conclude that 7S/11S covalently conjugated with G/F mixes during dry heating. Similarly, SDS-PAGE findings for 7S/11S with G/F conjugates were described by [44], which suggests that soy protein subunit (7S/11S) cross-linking and the Maillard reaction among the amino groups of 7S and 11S and the carbonyl residues of glucose and fructose had actually taken place.

### 2.4. Surface Hydrophobicity (H_0_)

Protein conformation modifications may be reflected in surface hydrophobicity (H_0_) which is defined as the quantity of hydrophobic groups that are exposed on a protein molecule’s surface. Surface hydrophobicity is strongly associated with the functional characteristics of proteins in food matrixes and gels [45]. Surface hydrophobicity could represent a static element, and elasticity may be an unpredictable variable in the outermost properties of proteins with the extension of the reaction period.

**Figure 6:**
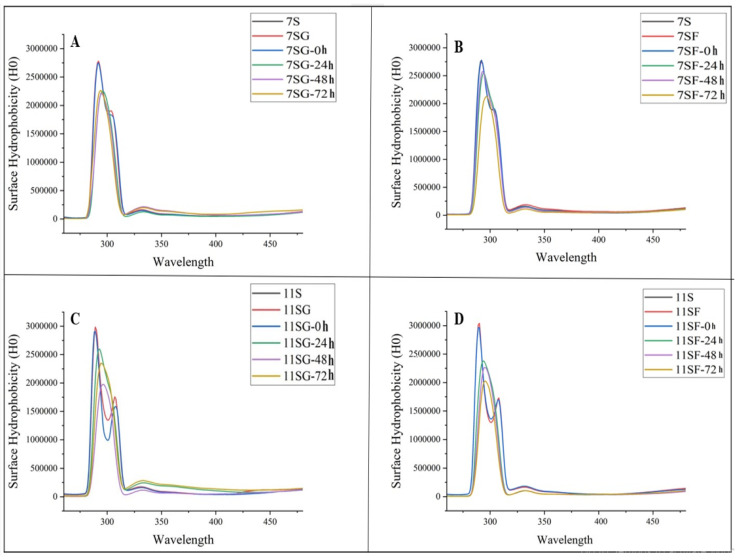
Effects of reaction time on surface hydrophobicity of 7S/11S with G/F conjugates: (**A**) conjugation of 7S with glucose; (**B**) conjugation of 7S with fructose; (**C**) conjugation of 11S with glucose; and (**D**) conjugation of 11S with fructose.

Figure 6 demonstrates that the samples’ H_0_ considerably increased (*p* < 0.05) in comparison to the untreated 7S/11S and the conjugation of 7S/11S with G/F (0 h) sample. It also displays a rising trend before a major decrease. According to the findings, the Maillard process caused the surface hydrophobicity of soy proteins to increase, unfolding its molecular structure and exposing its hydrophobic group. Furthermore, it was observed that there was a consistent fluctuation in both surface flexibility and hydrophobicity, indicating a potential correlation between these two factors. Previous studies have shown that protein denaturation increases surface hydrophobicity, while the development of aggregates in gel-type food matrices reduces it. This observation exhibited similarity to previous studies conducted by [46,47]. According to [48], an increased duration of the reaction time resulted in a higher exposure of hydrophobic groups. These hydrophobic groups may have undergone repolymerization via hydrophobic activity, leading to a decrease in hydrophobicity.

### 2.5. CD Analysis

**Figure 7:**
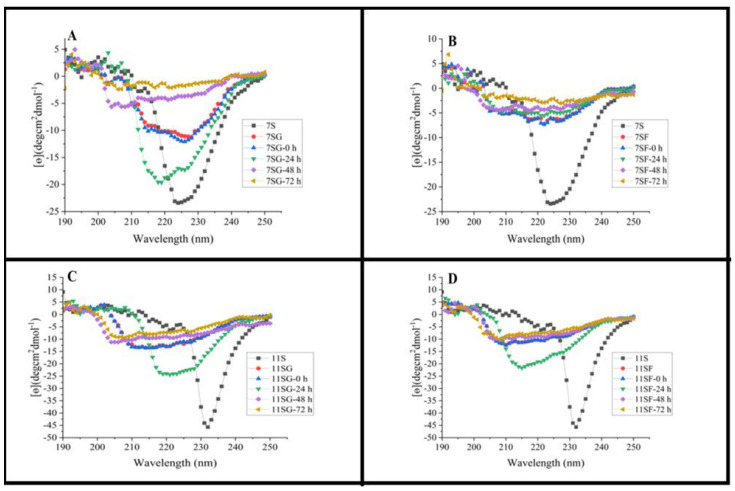
Circular dichroism spectra of 7S/11S with G/F at 70 °C: (**A**) conjugation of 7S with glucose; (**B**) conjugation of 7S with fructose; (**C**) conjugation of 11S with glucose; and (**D**) conjugation of 11S with fructose.

Modifications in the secondary structures that comprise the untreated 7S and 11S, as well as their conjugates (7S/11S with G/F) produced via the Maillard process, were found by employing the far-ultraviolet CD spectra (Figure 7). The presence of a β-sheet configuration in the 7S and 11S structures was suggested due to the negative shoulder area peak at 205 nm, while the α-helix structure’s negative Cotton effect may have led to the negative cleft at 205 nm. The CONTIN/LL algorithm that was made available by Jasco Corp. and was derived from the process outlined by [49] was utilized to determine the secondary structure. Table 1 and Table 2 display the predicted secondary structure composition. It was discovered from Table 1 and Table 2 that 7S has a 41.16% unordered structure, a 9.6% α-helix, a 43.53% β-sheet, and 15.26% β-turns. The α-helix composition of 7S is considerably altered due to a combination of glucose and fructose, while the remaining secondary structure remains mostly unchanged. According to reports, polysaccharides attached to the ε-amino group within the α-helix region are responsible for the reduction in the α-helix concentration [50]. There was additionally an observation of α-helix structure reduction in isolates of soy protein conjugated with glucose; the reduction of the secondary structure was attributed to either heating-induced unfolding or the binding of glucose and fructose. Nevertheless, when it comes to 11S, there was a significant increase in the α-helix abundance when it was conjugated with glucose and fructose; however, the other secondary structures stayed unchanged [51]. In summary, the α-helix content of 7SG may have been reduced due to thermal unfolding and grafted dextran, resulting in the transformation of β-sheet, as well as unorganized structures.

The percentage of α-helices and their conjugates’ unorganized structures was greatly increased and then fell, whereas the percentage of β-sheet and β-turns typically declined and then grew as the Maillard reaction involving 7S/11S and G/F proceeded [52]. Previously, it was clarified that Dex’s entry into the protein chain might be ascribed to molecular becoming entangled and various interactions like those of Van der Waals and hydrogen bonds between Dex and the charged group, i.e., phosphatidylserine within the phosvitin core. Nonetheless, our investigation suggests that glycoprotein aggregation is responsible for the enhanced β-sheet and lowered α-helix amount found in 7SF after 24–72 h of heat treatment. Within the treatment period, the β-helix portion of 11S with G/F was first reduced and then raised, whereas the β-sheet percentage first fell and then increased. The denaturation of proteins may be connected to the rise in the number of unordered molecules [53]. Consistent with our fluorescence study, the data here showed that a 24–72-h duration of therapy may have resulted in a greater rate of unfolding compared to protein aggregation.

### 2.6. Thermal Properties

Liu et al. [48] reported that the thermal stability of individual proteins and their mixes in different food gels may be enhanced via the Maillard reaction that occurs among monosaccharides and proteins. Differential scanning calorimetry (DSC) is a useful tool for determining a sample’s thermal stability because it can identify variations in the sample’s heat flow increases during temperature changes.

**Figure 8:**
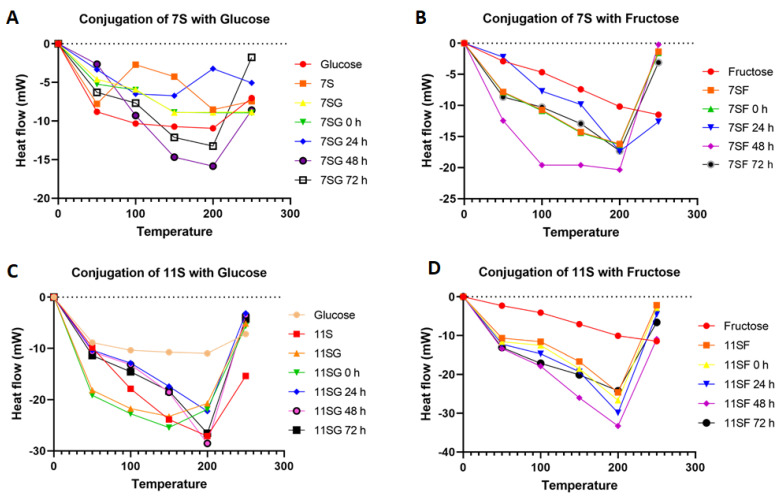
Thermal stability patterns of heated 11S/7S with G/F over different reaction times: (**A**) conjugation of 7S with glucose; (**B**) conjugation of 7S with fructose; (**C**) conjugation of 11S with glucose; and (**D**) conjugation of 11S with fructose.

Depending on the DSC parameters of the 7S/11S with a G/F combination and the conjugates that were added during different incubation periods, as shown in Figure 8, we observed that the denaturation transition of the conjugates was significantly greater compared to that of the mixture. The denaturation temperature of the conjugates’ increases varied from 50 °C to 200 °C throughout 24 h of incubation. Increased denaturation temperatures led to improved thermal stability. The results showed that the thermal conductivity of the 7S/11S with G/F combination was greatly enhanced via the Maillard process, which may be explained by the suggestion of [48] that glycation may enhance the steric repulsive interactions that occur among protein molecules. The temperature of 7S/11S with G/F conjugates rose from 210 °C in the incubation period, while the onset temperature for combined 7S/11S and G/F was 160 °C. Our results supported earlier relevant studies’ hypotheses that protein glycation could enhance a substance’s thermal resistance [40].

## 3. Conclusions

The Maillard reaction occurs when proteins and sugars are heated together. The results of this investigation showed that the Maillard reaction changed the soy protein 7S/11S isolates’ conjugation with G/F to a looser and more uniformly distributed spatial protein structure. The size of the sugars’ carbohydrate chains might be increased to reduce the soy peptides’ grafting rate, as well as the surface charge. Due to structural alterations, 7S/11S with G/F conjugates showed improved functional characteristics over single 7S/11S proteins, particularly in terms of stability and water-holding capacity. The soy protein isolate’s hydrolysis produces more low-molecular-weight fragments of peptide and enhances the protein’s solubility and capacity to retain water. In the near future, bioactive compounds may be encapsulated in walls made of 7S/11S with G/F conjugates, which could improve the compounds’ functional characteristics.

## 4. Materials and Methods

### 4.1. Chemical Reagents

Analytical-grade chemicals were purchased and used in this investigation.

### 4.2. Isolation and Separation of 7S and 11S from Soy Protein Isolate

The isolation of 7S and 11S from soy protein isolate was conducted by following our previous study [24] (Ud Din et al., 2021).

### 4.3. S/11S-G/F Conjugates Preparation

The method used for coupling in this study was based on the procedure established by previous researchers [25,26] with some modifications. Dried 7S and 11S protein and G and F were mixed in a 1:1 mass ratio of 0.01 M of phosphate buffer solution (PBS) at a pH of 7.2–7.4 and stirred for 2 h at room temperature (25 °C). Then, the mixture was rehydrated overnight at 4 °C. The resulting solutions were lyophilized, milled, and incubated at 70 °C for 0, 24, 48, and 72 h. The 7SG, 7SF, 11SG, and 11SF conjugates were extracted after the mentioned times and kept at 4 °C for further analysis.

### 4.4. Analysis of the Grafting and Browning Degree

For the analysis of the graft score (GD), the method of [27] was used after some minor modifications. A mixture of 40 mg of OPA, 1 mL of methanol, 25 mL of 0.1 M sodium borate solution (pH 9.75), 100 μL of *β*-mercaptoethanol, and 2.5 mL of 20% SDS (*w*/*v*) was used for the OPA mixture. After dilution with 50 mL of deionized water, 4 mL of the OPA mixture was added to 200 μL of a 0.4% sample solution, and the mixture was incubated at 35 °C for two minutes. The absorbance values of the samples were measured at 340 nm using a spectrophotometer (Shimadzu, Kyoto, Japan). In the control, the sample was replaced with distilled water. The grafting degree (GD) was determined as follows:

GD (%) = [(A_0_ − A_t_)/A_0_] × 100%

where A_0_ is the absorbance of the 7S/11S and G/F conjugates (0 h) samples, and A_t_ is the absorbance of the sample after reacting. The browning degree (BD) was expressed according to the absorbance measured at 420 nm. Before determination, the samples were diluted to the protein concentration of 2 mg/mL with the 0.1% SDS solution.

### 4.5. Analysis of Sulfhydryl Content on the Protein Surface

According to a modified method of [28], the total surface sulfhydryl content on the protein surface was analyzed. Samples were dissolved in PBS containing 1.0 mM of EDTA and a pH 8.0 buffer solution at a concentration of 20 mg/mL. Therefore, 50 µL of 1.0 mM 5,5′-dithiobis-(2-nitrobenzoic acid) (DTNB, Ellman’s reagent) solution was mixed with the sample solution (500 µL) and buffer solution (2.25 mL). Subsequently, the reaction solution was left protected from light at 25 °C for 20 min. A 412 nm wavelength was used to measure the absorbance against a blank of 500 µL of reaction buffer solution. All experiments were accomplished in triplicate. The following formula was used to calculate the surface sulfhydryl group content [SH]:

$$[\mathrm{SH}]\;(µ\mathrm{mol}/g)=\frac{10^{6}\times A_{412}\times D}{13,600\times C}$$

where D is the dilution factor of the sample solution, 13,600 is the extinction coefficient, C is the protein concentration (mg/mL), and A_412_ is the absorbance at 412 nm.

### 4.6. Confirmation of Soy Protein Isolate–G/F Conjugates

According to [29], an SDS-PAGE analysis was performed. A 5% stacking gel and a 12% separating gel were used. A sample solution with a 5 mg/mL mass concentration was mixed with a 5x SDS loading solution at a ratio of 4:1 and heated in a water bath for 5 min. After cooling at room temperature, 10 µL of the solution was loaded into each well. Electrophoresis was performed at a constant current of 80 V for 20 min and then at 120 V for about 1 h; the shaker was set at 60 rpm for 2 h.

Protein staining: Following electrophoresis, the gel was treated with Coomassie brilliant blue R250 (0.05%, *w*/*v*) in a solution of methanol–acetic acid–water (25:10:65 *v*/*v*/*v*) for staining. The gel was then destained in the same solution without the dye.

Sugar staining: The gel strips were submerged in the cyclic acid solution for a duration of 12 h, followed by rinsing with a trichloroacetic acid solution for 2–4 cycles, each lasting 2 h. The strips were thereafter subjected to Schiff’s reagent under dark conditions for a duration of 16 h, followed by immersion in a 5% acetic acid solution until the appearance of a pink glycoprotein band. The gels were subsequently scanned using the Molecular Imager Chemi Doc XRS+ equipment from Bio-Rad (Hercules, CA, USA) and densitometric analysis was conducted using the Image Lab software, 3.0.1 also from Bio-Rad, USA.

### 4.7. Surface Hydrophobicity (H_0_)

The proteins’ surface hydrophobicity (H_0_) values in the samples were assessed using 1,8-anilinonaphthalenesulfonate (ANS) as the fluorescent probe. A 5 mL sample of protein solution at various concentrations (0.0125, 0.025, 0.05, and 0.1 mg/mL) was generated using a 0.01 mol/L phosphate buffer with a pH of 7.0. Next, a volume of 20 μL of ANS solution (8.0 mmol/L, pH 7.0) was introduced into the protein solution. The fluorescence intensity of the resulting mixture was then measured after thorough mixing for 5 s and allowing it to stand undisturbed for 15 min at room temperature in a dark setting. The excitation wavelength was configured to 300 nm, the emission wavelength was set to 450 nm, and the slit width was adjusted to 5 nm. The fluorescence intensity was measured and plotted against the concentration of the protein solution. The slope of the regression line, which exhibited a strong linear relationship, was chosen as the protein’s surface hydrophobicity index (H_0_).

### 4.8. Measurement of Circular Dichroism Spectra

According to [30], the modification in the secondary structure of the protein was investigated using circular dichroism (CD) spectroscopy (Bio-Logic Science Instruments SAS, Seyssinet-Pariset, France) in the far-ultraviolet region. Weighed samples were dissolved in a 0.01 mol/L phosphate buffer (pH 7.0) to a concentration of 0.4 mg protein/mL. Afterward, for 20 min at 20 °C, any unsolvable residues were removed by centrifuging at 12,000 g. The final concentration of the proteins used for CD analysis was 0.2 mg/mL. We used an MOS-500 (Bio Logic, Seyssinet-Pariset, France) to scan between 200 and 250 nm. The experiments were conducted at 20 °C. The optical length of the sample cell was 1 mm, the sensitivity was 100 mdeg/cm, the scan speed was 100 nm/min, and the resolution was 0.1 nm. Each scan was performed in triplicate, and the mean was calculated from the experimental data. The spectra were analyzed using software Ver.2.5 via https://bestsel.elte.hu/index.php (accessed on 15 July 2023) to calculate the *α*-helix, *β*-fold, *β*-turn, and random curl percentage.

### 4.9. Thermal Properties

To evaluate the thermal properties of conjugates, differential scanning calorimetry (DSC) (TA Instruments Q2000, New Castle, DE, USA) was used. A nitrogen atmosphere was used to load approximately 3–5 mg of conjugate powder into a sample pan, while an empty sample pan served as a reference. The temperature was raised from 30 °C to 250 °C at a steady rate of 5 °C/min

### 4.10. Statistical Analysis

All the samples were measured in triplicate, and the results were expressed as means ± standard deviations (SDs).

## Figures and Tables

**Table 1:** Secondary structural content of 7S and glucose/fructose conjugates treated at 70 °C estimated from circular dichroism spectra.

Sample	*a*-Helix	*β*-Sheet	*β*-Turn	Other
7S	9.6 ± 0.15 ^b^	43.5 ± 2.07 ^a^	15.2 ± 1.25 ^c^	41.1 ± 3.29 ^bc^
7SG	5.6 ± 0.14 ^b^	42.2 ± 2.07 ^a^	14.2 ± 1.25 ^c^	42.1 ± 3.29 ^bc^
7SG-0 h	5.9 ± 0.13 ^ac^	42.3 ± 0.51 ^b^	14.5 ± 0.05 ^bc^	43.1 ± 0.55 ^a^
7SG-24 h	2.7 ± 0.09 ^d^	42.0 ± 0.41 ^bc^	14.6 ± 0.17 ^d^	42.7 ± 1.00 ^ac^
7SG-48 h	0.9 ± 0.08 ^ce^	42.7 ± 0.81 ^c^	14.6 ± 0.06 ^c^	42.5 ± 0.96 ^d^
7SG-72 h	0.6 ± 0.08 ^ab^	42.1 ± 0.25 ^d^	14.4 ± 0.11 ^bc^	42.3 ± 0.32 ^e^
7SF	11 ± 0.28 ^b^	41.9 ± 0.98 ^e^	14.5 ± 0.21 ^d^	43.6 ± 0.95 ^cb^
7SF-0 h	11 ± 0.28 ^b^	41.9 ± 0.98 ^e^	14.5 ± 0.21 ^d^	43.6 ± 0.95 ^cb^
7SF-24 h	8.6 ± 0.31 ^d^	42.8 ± 0.45 ^f^	14.5 ± 0.23 ^e^	42.6 ± 0.68 ^e^
7SF-48 h	7.5 ± 0.48 ^e^	42.8 ± 0.37 ^af^	14.9 ± 0.64 ^b^	42.1 ± 0.66 ^f^
7SF-72 h	2.1 ± 0.12 ^ad^	42.6 ± 0.55 ^ef^	15.0 ± 0.43 ^ab^	42.3 ± 0.35 ^a^

Every value is the mean of three replicates ± the standard deviation [SD]. Values that do not share the same letter (superscript) are significantly different.

**Table 2:** Secondary structural content of 11S and glucose/fructose conjugates treated at 70 °C estimated from circular dichroism spectra.

Sample	*a*-Helix ± SD	*β*-Sheet ± SD	*β*-Turn ± SD	Other ± SD
11S	16.3 ± 1.12 ^f^	42.3 ± 0.77 ^ab^	14.6 ± 0.05 ^a^	43.0 ± 0.72 ^cd^
11SG	38.5 ± 2.22 ^c^	42.5 ± 0.21 ^a^	14.6 ± 0.10 ^b^	42.9 ± 0.17 ^d^
11SG-0 h	38.5 ± 2.22 ^c^	42.5 ± 0.21 ^a^	14.6 ± 0.10 ^b^	42.9 ± 0.17 ^d^
11SG-24 h	59 ± 2.56 ^a^	41.7 ± 0.71 ^bc^	14.1 ± 0.56 ^c^	44.0 ± 1.10 ^ab^
11SG-48 h	35.3 ± 0.98 ^d^	41.9 ± 0.55 ^d^	14.1 ± 0.51 ^d^	43.9 ± 0.96 ^a^
11SG-72 h	35.8 ± 1.45 ^d^	41.8 ± 0.56 ^ac^	14.2 ± 0.65 ^ab^	43.8 ± 0.98 ^e^
11SF	57.1 ± 1.87 ^a^	41.7 ± 0.15 ^de^	14.6 ± 0.17 ^bc^	43.5 ± 0.25 ^d^
11SF-0 h	57.1 ± 1.87 ^a^	41.7 ± 0.15 ^de^	14.6 ± 0.17 ^bc^	43.5 ± 0.25 ^d^
11SF-24 h	21.3 ± 2.33 ^e^	42.1 ± 0.87 ^d^	13.9 ± 0.41 ^ae^	43.9 ± 1.15 ^d^
11SF-48 h	47.7 ± 2.54 ^b^	42.1 ± 1.18 ^ad^	14.6 ± 0.26 ^d^	43.2 ± 1.28 ^f^
11SF-72 h	38.3 ± 1.99 ^c^	42.3 ± 1.21 ^a^	14.8 ± 0.71 ^ad^	42.8 ± 1.91 ^e^

Every value is the mean of three replicates ± the standard deviation [SD]. Values that do not share the same letter (superscript) are significantly different.

## Data Availability

The data presented in this study are available upon request from the corresponding author. The data are not publicly available due to part of the data undergoing in-depth research.

## List of Abbreviations

ANS	1-anilino-8-naphthalene sulfonate
AP	Ammonium persulfate
BA	Bis N,N-Methylene-bis-(acrylamide)
BG	Browning degree
CD	Circular dichroism spectroscopy
DTNB	5,5′-dithiobis-(2-nitrobenzoic acid)
DSM	Defatted soybean meals
DSC	Differential scanning calorimeter
EDTA	Ethylene diamine tetraacetic acid
F	Fructose
FI	Fluorescence intensities
G	Glucose
GD	Grafting degree
G250	Coomassie brilliant blue R-250
H_0_	Surface hydrophobicity
kDa	Kilodalton
Liq	Liquid
Mw	Molecular weight
NEM	N-ethylmaleimide
OPA	o-phthaldialdehyde
pI	Isoelectric point
SB	4× Laemmli sample buffer
SBS	Sodium bisulfite
SDS-PAGE	Sodium dodecylsulphate polyacrylamide gel electrophoresis
SH	Sulfhydryl content
SPI	Soybean protein isolate
Tris	Tris-(hydroxymethyl) amionmethane
TEMED	N, N, N′, N′-Tetramethyl ethylere-diamine
UV	Ultraviolet
2-ME	2-mercaptoethanol
